# Obesity Prevention: A Systematic Review of Setting-Based Interventions from Nordic Countries and the Netherlands

**DOI:** 10.1155/2018/7093260

**Published:** 2018-04-01

**Authors:** Jacqueline Panter, Pernille Tanggaard Andersen, Arja R. Aro, Anastasia Samara

**Affiliations:** Unit for Health Promotion Research, University of Southern Denmark, Esbjerg, Denmark

## Abstract

**Aim:**

Effective evidence-based interventions have an important role in obesity prevention. Our aim was to present a qualitative synthesis of setting-based health promotion interventions on obesity, from Nordic countries and the Netherlands.

**Methods:**

A systematic review of the literature was completed for studies in the community, schools, and worksite, with BMI as an outcome. A descriptive analysis was completed for all full-text articles meeting the inclusion criteria.

**Results:**

Thirty-three articles were identified: 7 whole of community, 3 worksite, and 23 school-based interventions. The studies were largely quasiexperimental in design (21/33), with follow-up from 4 months to 8 years. The explicit use of theory was not featured in many of the studies (20/33). No consistent direction for BMI change could be identified in the whole of community interventions (2/7 positive, 2/7 negative, and 3/7 no effect) and no effect for worksite (3/3 no effect) or many of the school-based interventions (1/23 negative, 4/23 positive, 15/23 no effect, 1/23 BMI significant increase only for control group and 3/23 no data available).

**Conclusions:**

There is a need to prioritise interventions with study designs of high quality, theory, and a participatory approach, for optimal implementation and evaluation of obesity prevention interventions.

## 1. Introduction

The rise in obesity in the past several decades has been dramatic worldwide, particularly in the Western world. According to data from 2016, WHO reports that the Nordic countries and the Netherlands have similar rates for overweight and obesity (people with a BMI ≥ 25 kg/m^2^) that vary within 4 percentage points; from Denmark with the lowest 55.4% to Iceland with the highest 59.1%. These rates are lower than many Western countries (such as Canada, USA, Australia, New Zealand, UK, France, Spain, Greece, and the Middle East). Similar are the results for obesity (lowest for Denmark with 19.7% and highest for Norway with 23.1%). These rates are also lower than many Western countries, as mentioned above (excluding France) [1]. However, these rates are still considered high and suitable initiatives are needed in order to reduce them.

Nordic countries and the Netherlands are highly regulated welfare states. They are also countries in geographical proximity with similarities in their societies such as economic and social policies. Therefore, these countries can apply similar initiatives and can be compared with each other. A regional focus allows for a more targeted analysis and provides results and conclusions that can benefit at the regional level [2, 3]. In addition, these countries prioritise public health and have been progressive in implementing health promotion strategies, addressing the lifestyle determinants of obesity at a national level. These strategies have included the provision of national nutrition and physical activity (PA) guidelines and associated campaigns, positive changes to school curriculum, and, in Sweden and Finland, the provision of free school lunches and financial incentives for health promotion at the worksite. Nordic countries, in recent years, have also seen a general shift of responsibility for obesity prevention interventions to the local municipality level [2]. Therefore, they can provide valuable information about health promotion in relation to obesity, compared to other European countries or even serve as a model/example for the other countries.

Well planned, implemented, and evaluated setting-based interventions are paramount in measuring the success, future directions, and financial commitment of interventions for obesity prevention. Bottom-up approaches enable taking into account the needs of the intervention participants and the characteristics and resources of the context. This makes interventions more feasible to implement and more salient to the participants; these aspects increase the sustainability of desired outcomes. Research evidence supports the bottom-up approach since it can help overcome barriers of required change [4].

Multilevel approaches that involve the environment of the individual are highly significant for fighting the obesity epidemic, as environmental factors are often a root cause of obesity [5–7]. Integrated, multilevel approaches are needed instead of single level interventions targeting separate determinant levels [8]. These approaches involve intervention components that create a “healthier environment,” such as school curricula and built environment changes, in addition to traditional approaches such as individual counselling and screening that have a limited impact. An example of the environmental approach in childhood obesity prevention could be changing the classroom interior, to allow physical activity in all lessons, instead of only during physical exercise lessons. Monitoring this practice showed clear decrease of obesity among the school children in Finland in the school setting—with results in obesity decrease [9].

The school setting is equally important both for children and their parents, especially as this setting is where children spend a large amount of their time during the day. Schools are places where children consume one or more meals per day. They are places where canteens, vending machines, and restaurants are often available which can negatively influence children's eating habits. In addition, children spend a lot of time sitting in school. Physical education, as well as the provision of available spaces for play and activities, can improve their PA levels. School-based interventions have provided evidence for effectiveness of childhood obesity prevention [10].

Similarly, the worksite setting is of high importance, due to the considerable amount of time most adults spend at work. There are also opportunities to improve the worksite with exercise facilities, such as access to gyms, and with improved access and availability of healthy food provided in restaurants, canteens, or as snacks that can encourage people towards healthier habits.

Community-based interventions are also very important because they can create a healthier environment for people to live in, through parks, policies on fast food, cycling and jogging tracks, awareness campaigns, and so on. Therefore, they can be very powerful for affecting diet and PA habits in a community [11].

A thorough review of community-based interventions, addressing obesity prevention in the Netherlands through an equity lens, reported that these interventions have impacted socioeconomic inequalities in health behaviour positively and negatively [3]. A recent review of lifestyle interventions implemented in European schools including five studies from Norway, Iceland, Sweden and the Netherlands revealed limited studies which reported a reduction in Body Mass Index (BMI) [12]. Moreover, results from a scoping review of 71 community-based interventions against childhood obesity in Europe revealed limited studies where BMI was measured as an outcome [13].

Furthermore, reviews of health promotion interventions implemented at the worksite, globally and in Nordic countries, have found that the majority of studies utilised the worksite as a convenient setting to implement interventions targeted at individual behaviour change, rather than use a setting-based, multilevel approach including changes to the worksite environment [14–16]. To the knowledge of the authors, no review has been identified which has given an overview of all setting-based obesity prevention interventions, implemented in Nordic countries and the Netherlands.

Different components that define the quality of a study such as representativeness, randomisation process, comparability of chosen intervention and control groups, attrition rate, and spillover effect/attributability to intervention also need to be considered. The quality of a study affects highly the outcome, and a low-quality study might obscure the impact of the intervention otherwise evidenced. Another important element in evaluating the quality of interventions and an integral part of designing and planning complex interventions is the use of theory. This has also been acknowledged by the British Medical Research Council and forms part of its guidance [17].

The aim of this review was to identify, synthesise, and evaluate the quality of interventions including environmental components based in the in settings from Nordic countries and the Netherlands, aimed at preventing obesity where BMI was measured and reported as an outcome.

## 2. Methods

The review of the literature was completed systematically, guided by the PRISMA (Preferred Reporting Items for Systematic Reviews and Meta-analyses) statement [18], with guidance for the search strategy from a previous review of whole of community interventions [11]. The eligibility criteria for the studies selected was defined using PICO (Participants, Intervention, Comparison, Outcome).

### 2.1. Types of Participants

Interventions targeted all age groups, living in either the Netherlands or the Nordic countries: Denmark, Finland, Iceland, Norway, and Sweden, regardless of socioeconomic status (SES). The Nordic countries were included in the review based on geographical and cultural similarities. The Netherlands were included due to the similarities of their historical welfare model to that of the Nordic countries in general [19]. Studies selected included participants that were otherwise healthy, for example, not obese or with a preexisting condition, for example, hypertension.

### 2.2. Types of Interventions

We chose interventions in the community, school, and worksite setting with at least one environmental component. The community is considered a setting as much as the worksite and school [20]. Planned community-based interventions targeting the weight status of a population, characterised along geographical boundaries, such as cities, villages, or regions, are commonly defined as “whole of community” interventions [21]. An environmental component was defined as any effort in the setting that did not include individual-based strategies such as counselling for individuals, web-based computer-tailored feedback, or individual counselling. Such components were, for example, school curriculum changes, infrastructure and built environment, policies, restaurants, and so on. Since all studies had at least one environmental component, they were all socioecological models [22].

No restrictions were made to length of follow-up. English language studies published in the literature up to and including April 2016 were included in the review. Hospital-based clinical interventions or those primarily based in the primary care setting were excluded. Furthermore, worksite-based interventions were excluded if the target group was deemed too specialised and not representative of the general population of employees, for example, one professional group only. If there was more than one article referring to different follow-up points, the longest follow-up was chosen as the included article.

### 2.3. Types of Studies

All intervention study designs other than purely qualitative were included.

### 2.4. Outcome Measures

Interventions where the outcome was obesity or chronic disease prevention and where BMI was measured and reported as either a primary or secondary outcome were included. Studies that measured only behavioural outcomes including dietary or PA levels were excluded.

### 2.5. Search Strategy

A thorough search of the databases Medline and Embase through the Ovid search strategy was completed for articles published until April 2016 (Table 1). Additional sources included articles sourced from reference lists of review articles, identified through the original Ovid database search strategy, and from a search of databases: health evidence reviews and the cochrane database. Other sources by snowballing included articles identified from screening references of full-text articles. One researcher essentially performed the search and screening. After duplicates were removed, records were screened by title and abstract by the selection criteria, before full-text articles were identified. Reasons were provided for why articles were excluded by full-text. Full-text articles were reviewed by all authors.

### 2.6. Extracted Information

Studies meeting the inclusion criteria by full-text were classified by setting and country. Data were extracted independently by two researchers. A descriptive analysis of the studies involved extracting information including study design, participants, gender as a percentage of females, mean age (SD), total follow-up, measure of SES (education), and if a theoretical base (data not shown) was used for the intervention design and implementation. Further assessment of the outcomes of each study was reviewed with information extracted including the outcomes measured, description of the study population units, response rate and loss to follow-up, randomisation used, selection process for setting or community of choice, summary of intervention implemented, and lastly the outcome related to BMI. Where information was insufficient regarding baseline data or intervention design for a particular article, additional reference articles were sourced from respective reference lists or via a search in Pubmed by study name. Lastly, some additional estimated calculations were made by the authors for the response rate, lost to follow-up, and gender, based on the information available from the articles.

### 2.7. Quality Assessment of Studies

An analysis of the methodological quality of the studies was then completed using a quality assessment tool [23], previously used by the authors of a review of lifestyle interventions in the Netherlands [3, 23]. The quality assessment was performed independently by two researchers. Representativeness was considered as a response rate of 60% or more in samples randomly recruited from the study population, or that the study showed otherwise to be representative of the population [3]. In the case of the whole of community interventions, we considered the participants within the community (random selection) as units to determine representativeness. For the other setting-based interventions, we considered the schools or worksites as units to determine representativeness (not the children or students). We also considered the choice of setting/community (e.g., convenience, volunteering, and participation in existing programs) in order to judge whether a sample was representative or not.

Finally, comparability was difficult to determine, especially if some but not all baseline characteristics were similar. Available data were assessed when a study included BMI in their baseline description and were deemed noncomparable if there were differences in BMI, even if there were no differences in other characteristics. In addition, we considered the baseline characteristics comparable, if the intervention and control group were matched or selected based on similar characteristics, such as SES.

### 2.8. Data Synthesis

Data is presented by setting in the following order: whole of community, worksite, and school. Data were not pooled or regrouped based on specific characteristics but are presented and discussed as separate settings. Pooling or regrouping of the data was not possible due to the heterogeneity of the studies.

## 3. Results

**Figure 1:**
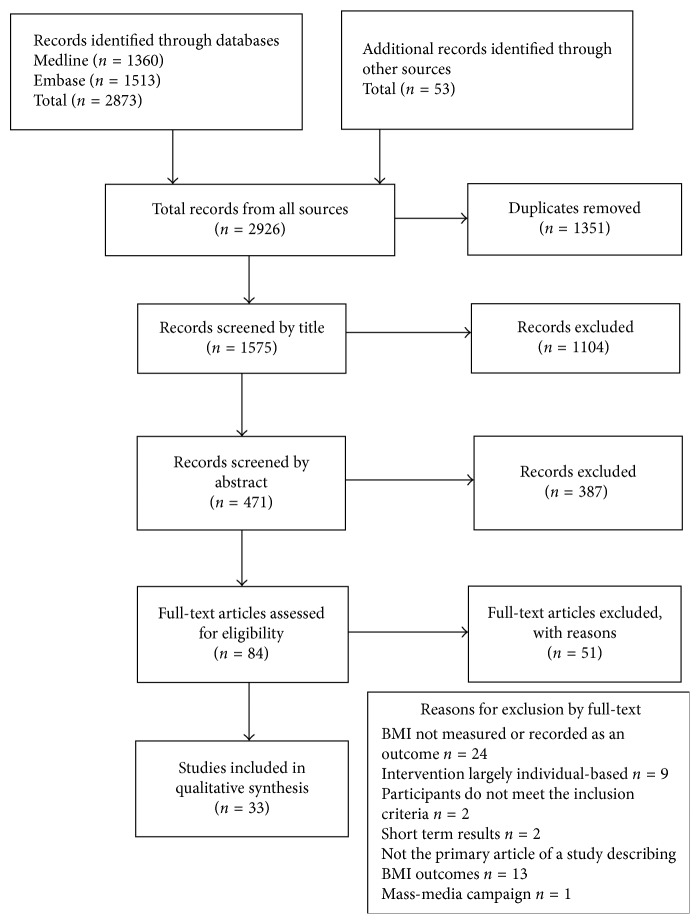
Study identification, screening, and eligibility, guided by PRISMA.

The literature screening process is presented in Figure 1. The major search revealed 2873 articles, and additional sources revealed 53 more articles. After removal of duplicates, 1575 were available for screening. Screening by title and abstract led to 84 full-articles; of those, 33 were finally included for analysis [24–56].

Table 2 shows the descriptive characteristics and assessment of all three types of setting-based interventions, with Table 3 providing a summary of key characteristics of these setting-based interventions. Out of the total number of studies, seven were whole of community interventions [37, 39, 42, 44, 45, 52, 56], three were worksite-based interventions [34, 36, 43], and 23 were school-based interventions [24–33, 35, 38, 40, 41, 46–51, 53–55].

Weight prevention was a secondary outcome in five out of the 33 studies: changes in dietary habits [24], muscle development [55], health behaviour [28, 29], and increase in PA indicators [32]. These studies were all school-based interventions. Where required, some additional information was extracted, regarding theoretical constructs from additional articles related to the original studies [57–64] (data are not shown).

### 3.1. Whole of Community Interventions

Among the whole of community interventions, two were pre-post studies without a control group [37, 44], and there was no cluster randomised design in any of them. All other studies were quasiexperimental [39, 42, 45, 52, 56]. The percentage of females ranged from 46.0% to 57.2%. There was no information for either education level or SES of the participants in 2 [37, 44] of the 7 interventions. BMI change for adults was measured in all studies. The total time of follow-up varied from three to eight years, and four interventions included a cohort (same individuals followed) and cross-sectional samples [37, 42, 44, 56].

All whole of community interventions focused on risk factors for cardiovascular disease (CVD) and involved individual and environmental components in the interventions. Five studies were multicomponent studies (three components and above) [37, 39, 45, 52, 56]. Six studies were interested in diet, PA, and other risk factors [37, 42, 44, 45, 52, 56]. One study was interested in PA only [39]. Two interventions had components related to worksites [39, 45] and one intervention to schools and worksites [37] even though BMI changes for children were not measured in this study. Environmental components such as awareness campaigns were available in all seven studies [37, 39, 42, 44, 45, 52, 56], organised activities at little or no cost in six studies [37, 39, 42, 45, 52, 56], food stores in four [37, 44, 52, 56], infrastructure in two [39, 45], and policy in two (smoking) [45, 52]. One study focused additionally on capacity building (at least as reported strategy) [37]. Only one [39, 59] of the seven whole of community interventions was explicitly theory-based and used a multilevel approach, and only one [45] mentioned a theory (data not shown).

Changes in BMI for the interventions are presented in Table 2 in a variety of ways, depending on the type of follow-up (wave of cross-sectional samples or cohort). Out of the seven whole of community interventions, two showed a significantly lower increase in BMI in the intervention (I) group compared to the control (C) group [39, 52]. One study showed a significantly higher increase in BMI in the I group compared to the C group [45], and another showed an increase in BMI in a before-after study (comparisons among cross-sectional samples) [44]. Three studies showed no difference (one before-after study with comparisons among cross-sectional samples [37], one with cross-sectional samples and a cohort [56], and one cohort [42]). For the one cohort in this category [42], the differences for I and C groups were not tested. In this category, for the study that included cross-sectional samples and a cohort [56], there were cross-sectional samples taken for both I and C groups. Only the I group cohort for the study showed a decrease in BMI over time [56].

### 3.2. Worksite-Based Interventions

All three worksite-based interventions were quasiexperimental and measured BMI change for adults. The percentage of female participants in two of the studies was 37.4% to 50.7%, whilst one intervention included 100% male drivers [36]. For one [36] of the 3 interventions, there was no information for either education level or SES of the participants. The duration of the total follow-up time varied from one to two years, and all were cohorts. Two interventions focused on CVD risk factors [34, 36] and one focused on body mass composition [43].

Two of the interventions had an individual component [36, 43] and one involved the families of the participants (educational sessions) [36]. One study was a multicomponent study [43] and one had only one component [36]. All three studies were interested in diet and PA [34, 36, 43]. Environmental components such as awareness campaigns were available in two studies [34, 43], canteens in two [34, 43], and organised activities in two studies [36, 43]. One [43, 60] of the interventions was theory-based and built upon a multilevel approach, including setting and individual levels (data not shown). All three worksite-based interventions showed no differences in the changes for the I and C groups. One of the interventions showed a decrease in BMI in the I and C groups (not significant) but the differences for the I and C groups were not tested in this study [34]. In another study, there was a decrease in BMI in the I group but not in the C group [43].

### 3.3. School-Based Interventions

Nine of the school-based interventions were cluster randomised [26, 30, 35, 38, 41, 46, 47, 49, 53] and one used historical controls [29]. All other 13 studies were quasiexperimental [24, 25, 27, 28, 31–33, 40, 48, 50, 51, 54, 55]. One of the school-based interventions was performed around the Dutch-German borders [48], and one other school-based intervention performed in Sweden was part of a cross-cultural study among eight European countries [31]. In all studies, the control group was chosen from either the same or a neighbouring area, region, municipality, setting, or with a similar SES. Females accounted for 39.7% to 60.1% of participants in the school-based interventions, excluding one intervention which included 100% girls [55]. In 16 [24, 25, 27–30, 32, 38, 40, 41, 48, 50, 51, 53–55] out of the 23 interventions, there was no information for either education level or SES of the participants or the parents of participants. All school-based interventions measured BMI change for children and adolescents. The duration of follow-up varied largely from four months to eight years. One study included both a cohort and cross-sectional samples [28] and one study included only cross-sectional samples [29]. Twelve interventions were focused on obesity [24–26, 30, 32, 38, 41, 47, 48, 54], six on body composition [31, 35, 40, 46, 53, 55], and the remaining five on CVD risk factors [27–29, 50, 51].

Fourteen school-based interventions were multicomponent studies [27–29, 31–33, 35, 38, 41, 46–50] and seven had only one component [24–26, 30, 51, 54, 55]. Five studies were interested in diet, PA, and other risk factors [28, 29, 31, 33, 50] and nine in diet and PA [27, 35, 38, 41, 46–49, 53]. There were six studies interested in PA only [30, 32, 40, 51, 54, 55] and three interested in diet only [24–26]. Five school-based interventions were also focused on the parents [28, 29, 31, 32, 38, 41, 47], two on the teachers' training [27, 40], and eight on both [28, 29, 33, 35, 46, 48–50]. Environmental components such as curriculum changes were available in 19 studies [27–32, 35, 38, 40, 41, 46–51, 53–55], food provision in nine studies [24–26, 28, 29, 35, 47, 50, 53], school-wide in five studies [31–33, 35, 48], awareness campaigns in four studies [31, 33, 47, 50], policy in four studies, community in three studies [31, 38, 48], capacity building in two [28, 29] and school infrastructure in two [27, 33]. Finally, four school-based interventions had only a curriculum component for increase in PA [30, 51, 54, 55], whereas three school-based interventions had only actions or changes related to diet performed [24–26].

Ten [31–33, 35, 38, 41, 46, 48, 49, 53] of the 23 school-based interventions were explicitly theory-based using a multilevel approach: like intervention mapping and whole school participation (data not shown). Most of them did not show any differences in BMI changes between the I and C groups [25, 26, 30–33, 35, 38, 40, 41, 46, 50, 51, 53, 55]. In one study, there were only differences for one of the two schools [28] (different intervention for each school); in another study, there were no differences for 2nd grade children [30] (however, no test for comparing the I and C groups was available in this study). One study showed a significantly higher increase in BMI in the I group compared to the C group [27]. Four studies showed a significantly lower increase in BMI in the I group compared to the C group [28, 30, 35, 48, 54]. Of these studies, one showed these differences only for females [35], one only for one of the two schools studied [28], and one only for 3rd grade children [30].

Finally, two studies showed a decrease in BMI in the I group [54] or in the I group for males but not for females [24]. Of these studies, Ask et al.'s study was the only study where there were no significant changes for the I group but there were for the C group [24] (however, no test for comparing the I and C groups was available in this study). Three studies did not have any available data as BMI mean or percent changes but changes in obesity rates. Therefore, they were excluded from comparisons [29, 47, 49].

### 3.4. Quality Assessment of Studies

Table 4 presents the quality assessment results of the included studies [24–56] with the use of supporting articles, where required [57, 59, 65–68]. None of the studies fulfilled all five criteria. There was only one study that fulfilled one criterion [39], 11 that fulfilled two [27–29, 31, 33, 44, 47, 48, 50, 51, 56], 14 that fulfilled three [24, 26, 30, 32, 37, 40–43, 45, 46, 53–55], and seven that fulfilled four criteria [25, 34–36, 38, 49, 52]. Credibility of data collection instruments was the only criterion that was fulfilled in all studies. Studies in which weight was self-reported [26, 28, 29] were also considered to fulfil the criterion. For representativeness, six interventions fulfilled this criterion, of which four were whole of community [37, 42, 52, 56], one worksite [36], and one a school-based intervention [26]. For randomisation, only nine interventions were randomised and these were all school-based [26, 30, 35, 38, 41, 46, 47, 49, 53]. In addition, two interventions included a random allocation of intervention or control groups among two [24] or three schools [25]. For the comparability, two community [45, 52], all three worksite [34, 36, 43], and 15 school-based interventions fulfilled the criterion [24, 25, 27, 28, 30, 32, 35, 38, 40, 41, 46, 49, 51, 54, 55]. For the attrition rate, four whole of community [37, 42, 44, 52], all three worksite [34, 36, 43], and 13 school-based interventions fulfilled the criterion [25, 31–33, 35, 38, 40, 48–50, 53–55]. Finally, for the attributability to intervention (likely that the observed effects are attributable to the intervention and not due to a contamination of the control group or to a concurrent intervention), there was very little available discussed in the articles. Among the whole of community, one fulfilled [45] and one did not fulfil [39] this criterion, two worksite-based interventions [34, 36] and one school-based intervention [29] fulfilled, and one [32] did not fulfil this criterion.

## 4. Discussion

To our knowledge, this is the first systematic review focusing on setting-based interventions on obesity prevention in Nordic countries and the Netherlands, which includes all age groups and types of settings. Results for BMI change showed no consistent direction for whole of community interventions (2/7 positive, 2/7 negative, and 3/7 no effect), no effect for worksite-based interventions (3/3), and no effect for many of the school-based interventions (1/23 negative, 3/23 positive, 15/23 no effect, 1/23 BMI significant increase for control group only, and 3/23 no data available). A quality appraisal showed that many studies poorly fulfilled criteria related to representativeness (25/33) and randomisation (20/33) or had no available information on attributability of the intervention (25/33). However, for comparability of baseline data (20/33) and attrition rates (20/33), evaluation was better.

Theoretical constructs are very important in research in general, for illustrating the associations between variables, the change process, and so on thus helping understand the interventions' mechanisms [69]. Especially in the field of behaviour change which is relevant to healthcare, ample work has been done in developing theories to guide studies. In this area, theory is very much needed and its value has often been underrecognised [70]. There are examples of interventions in obesity that showed results by using theory constructs' [71, 72].

Participatory interventions are also very important because they engage with people whose life-world and meaningful actions are under study. The target group should play a key role in planning, implementing, and adjusting the interventions. When constructing an intervention with a participatory dimension, it entails the mobilisation of people, feeling of empowerment, and self-efficacy. In the long run, this creates a better opportunity for sustainable solutions. The participatory research methods are geared towards planning and conducting the research process consequently, which means that the aim of the inquiry and the research questions develops out of the convergence of two perspectives: of science and of practice [73–75]. Research in the field of obesity has shown that participatory approaches are beneficial [76].

This systematic review illustrates that the studies in general used theory more as a background understanding, than for guiding the interventions, or for the discussion and interpretation of the results, and implications for further research. Several studies (18/33) did not use theory explicitly [24–27, 30, 34, 36, 37, 42, 44, 45, 47, 50–52, 54–56] and others used theories as the basis for choice of study design or approach (3/33) [35, 39, 43]. Some of the studies (7/33) [31, 32, 38, 41, 48, 49, 53] built on multicomponent and multilevel interventions such as intervention mapping (IM), socioecological theories or models, and models for individual behaviour; for example, theory of planned behaviour (TPB), social cognitive learning theory (SCT), and health belief model (HBM). Especially in the whole of community and school-based interventions, the background and theoretical frameworks were linked to theories of empowerment, participation, and whole-school participatory tailored approaches (5/33) [28, 29, 33, 40, 46]. Across the studies, there existed “a light way” of using theory. Theoretical framework was more often simply referred to, rather than something that was used concretely in problem formulation and as a basic structure in the studies. The lack of theoretical development and use in the field is an important finding. This can point to a greater need for the application of theory more specifically in future setting-based studies, thereby fulfilling the need for developing common standards and concrete theoretical basis for planning, implementing, and evaluating interventions within this field.

### 4.1. Whole of Community Interventions

The only available review on whole of community interventions on obesity [11] showed surprisingly that there are no available studies focusing primarily on obesity for adult populations. All of the whole of community interventions in our review had a broader scope, often targeting other risk factors for type 2 diabetes and CVD, beyond BMI. In addition, only one study [37] included a component on schools even though there were no measurements of change in risk factors for this young population. Although obesity is part of the causal pathway for these diseases, studies that do not primarily focus on obesity may risk having a weaker impact on BMI changes, due to the multiplicity of efforts directed towards other risk factors. Not only do other factors such as cholesterol, lipids, and blood glucose require a different timeframe to show meaningful changes, addressing alcohol and tobacco consumption also requires different strategies other than diet and PA.

Unfortunately, the vast majority of reviews on obesity and related risk factors are on children. We found only one systematic review [77] from 2003 on community-based interventions and type 2 diabetes that showed no significant improvements for BMI after intervention. Half of the whole of community interventions showed no change, and this did not seem to be related to a short duration of follow-up or to the use of a nonmulticomponent design. Other factors such as lack of complete implementation can explain the reduced impact in these studies. For two of these [37, 56], there was no information on implementation, and for one [42], full implementation was not achieved (problems with implementing PA due to lack of facilities and resistance to jogging) [42]. In addition, there was no information on implementation in the two studies that showed a favourable effect for BMI [39, 52]. However, in one of the studies [52], the authors considered that the reason for their success was the use of an integrative approach (participation of many actors such as organizations, sponsors, municipalities, etc.,) and the length of follow-up (five years).

Methodological issues can also reduce the strength of these studies and result in poor outcomes. One major issue is the use of a control group; without one, it is not possible to judge whether the changes are due to the project or due to other changes in that population. For the whole of community interventions, five were quasiexperimental. In addition, two of those used a cohort design, whereas the other three [42, 44, 56] also used cross-sectional samples, which increase the variance compared to following the same individuals over time. Randomisation is another concern, and it was not used in any of the whole of community interventions. However, randomisation in this setting is often difficult to implement. A review that analysed the effect of randomisation on the heterogeneity of studies showed that randomisation does not introduce a serious bias [20]. However, in all of the studies, there was an effort to choose a control group with similar characteristics to the intervention group, such as SES or a similar or neighbouring community. Apparently, this was not enough to create comparable groups, since 2 studies [45, 52] had comparable characteristics at baseline and 2 studies did not [39, 42]. Finally, attributability is a very important concern in health promotion studies due to possible ‘contamination' or other interventions implemented during the study period. Surprisingly, most of the studies including whole of community did not discuss this possibility even though it can significantly affect the outcomes.

### 4.2. Worksite-Based Interventions

We were only able to detect three studies, where most of those excluded were studies that used individual counselling. The included studies focused largely on CVD risk factors, similarly to the whole of community interventions. It is very difficult to draw any conclusions from this limited number of studies. However, the results identify a clear need for more obesity prevention studies with an environmental component, implemented in the worksite setting. There were no changes in BMI observed in any of these interventions (3/3), and this might be partially due to relatively short follow-ups (one to two years) and no multicomponent studies, which usually reflect a serious effort for change in the environment. For example, in only one of the studies [36], support from the family was considered. On the other hand, all studies had a control group, who were cohorts, showed mostly attributability and comparability even though there was no randomisation process employed in any of them. It has to be noted that one of the studies [43] made an effort to use randomisation; however, there was enormous resistance from the different worksites to be randomly selected as controls (out of 128 randomly selected worksites, 12 were finally studied). This is an example showing that methodological weakness can arise despite an effort from the researcher's side.

A review by Maes et al. [78] which included two of our studies [36, 43] categorised these studies as “moderate quality,” whereas many others were considered of “weak quality.” The quality appraisal included important criteria such as prior analysis of the needs of the worksite, integration of the activities in the management practices and daily working life of the enterprise, and theory-based intervention development. The authors also argued that the inconclusive effects on BMI change were mostly due to the lack of sufficient and high-quality studies. However, another review [14] showed a moderate effect on BMI. None of the above reviews can be directly comparable to ours because they also included studies with an individual component (e.g., dietician counselling) which might have resulted in a positive effect for BMI, at least for the intervention duration.

### 4.3. School-Based Interventions

Most of the studies showed no effect for BMI (15/23). A meta-analysis by Waters [20] showed that interventions in schools were effective for BMI, particularly for children 6–12 years old, and these were similar to the whole of community interventions for children [11]. However, the authors mentioned that there should be caution due to the small study bias and heterogeneity in the studies. Interestingly, nine out of 23 of the school-based interventions in our review were small studies (maximum three schools as I or C group) [24, 25, 28, 29, 46, 50, 51, 54, 55]. An interesting observation was that all of the single component interventions were in schools, but one [54] showed no changes in BMI (6/7), suggesting that such an approach is very limited for changing outcomes such as BMI. From the reasons described for initiation of these studies, it seems that in most cases, they had a very narrow scope related to the specific needs of the school. The duration of the intervention however did not seem to be related to BMI changes.

In some of the studies [24, 30, 35], there was no change in BMI (3/23) in the I group, and this is difficult to interpret. It would be tempting to consider this result as successful by suggesting that it stops the further BMI increase; however, there are other possibilities which actually do not reflect a successful programme: (1) there is no true effect or (2) ceiling effect because of normal weight of participants at baseline [35].

As for methodological issues, all school-based interventions were cohorts and had a control group but only nine were randomised studies. It is somehow reassuring that 15 studies showed comparability for baseline characteristics between I and C but that does not mean that studies should not use randomisation as a regular process. However, as mentioned previously, the process of randomisation is sometimes inhibited, as in one case where the schools participated only if they were considered as I schools [33]. Unfortunately, as for the other types of interventions, attributability was rarely discussed.

### 4.4. Limitations

Some major limitations of the articles included in our review are (1) very low reporting or discussion on attributability and SES, which is known to be associated with the effectiveness of a health promotion intervention, (2) unclear describing of results in some studies and some missing tests (comparison of changes in BMI between I and C which affected the way results are presented and possibly interpreted), and (3) reasons for choosing a region, municipality, worksite, or school such as practical (easy, already part of a project), with a very narrow scope in school-based interventions or initiation by local authorities which limit representativeness. Especially in the case of school-based interventions, representativeness was largely not fulfilled. However, in whole of community interventions, five were considered representative, showing most likely a more careful design view ‘heaviness' of these projects.

All of the whole community studies included awareness campaigns but few components such as infrastructure and policies. For the schools, 19 studies made changes to the curriculum; however, fewer interventions incorporated: improved infrastructure, policies, and school-wide or community level strategies. These results show a seeming lack in creating a healthier environment through broader and vaster changes, which is considered a major component of a health promotion study. In addition, very few studies implemented or at least described a capacity building process. Across the included studies, there was “a light way” of using theory and theoretical frameworks; these were mostly only referred to, without describing if and how they were used to guide the studies, select tools or interpret the findings.

One limitation of our study was not being able to organise the studies by length of follow-up or type of intervention components. This was due to the heterogeneity of studies. However, if we had decided to restrict our studies based on follow-up time, we would have to exclude a significant amount of studies that provide valuable information. In addition, grouping based on follow-up would have created many subgroup categories especially for the community and worksite-based interventions.

## 5. Conclusion

This review has provided an overview of obesity prevention interventions in seven whole of community, three worksite and 23 school-based interventions, implemented in communities of Nordic countries and the Netherlands, where BMI was reported as an outcome. This review was unable to demonstrate associations with BMI outcomes among these settings. However, it is very difficult to distinguish whether these results are due to the heterogeneity of the study designs, or due to poor quality in terms of design or implementation. In addition, initiation of a project especially in the school setting was often motivated by a very narrow scope related to the schools' needs, and not by an effort to test comprehensive strategies for obesity prevention.

There is a need to prioritise interventions that include study designs of high quality, the use of theoretical constructs to guide the studies, and a participatory approach for optimal implementation and evaluation of obesity outcomes. Use of theory at all levels of an intervention as well as promoting participatory approaches have been acknowledged to improve the effectiveness of different types of interventions, including obesity. Some suggested criteria for ‘good' theory in the area of behavioural change are: clarity of theoretical concepts, being explanatory, describing causality, testability, and so on. [79].

However, the future development of a published guideline on the complete, precise reporting of theory on different levels (process, implementation, and evaluation), even if possible in the obesity field, can help improve interventions. There are no explicit guidelines for setting-based interventions compared to clinical trials and interventions on diet and PA that focus on the individual. This leads to studies with no standard of quality based on defined criteria.

Guidelines should be created with an emphasis of criteria that can affect the study quality such as the ones we used in our review. An example of such criteria is a minimum period of follow-up based on evidence that shows how long it needs for obesity interventions to show a change in BMI, not factors such as political agendas. Another example is the consistent use of representative samples and randomisation as much as possible, instead of convenient and very small samples, especially for schools and worksites. Overall, it seems that presently there is not a serious commitment in preventing obesity through setting-based interventions and in particular in worksite interventions.

Commitment to further, more advanced research of settings-based interventions in these countries remains of vital importance, to secure and direct future investment for obesity prevention interventions.

## Figures and Tables

**Table 1:** Search strategy, medline and EMBASE via ovid.

(1) obesity.mp.
(2) childhood obesity.mp.
(3) overweight.mp.
(4) exp Obesity/pc [prevention and control]
(5) exp Cardiovascular disease/pc [Prevention & Control]
(6) (body mass index or BMI).mp.
(7) (Denmark or Danish or Dane$).mp.
(8) (Sweden or Swedish or Swede$).mp.
(9) (Norway or Norwegian$).mp.
(10) (Finland or Finnish or Finn$).mp.
(11) (Iceland or Icelandic or Icelander$).mp.
(12) (Netherlands or Dutch).mp.
(13) (Nordic or Scandinavia$).mp.
(14) communit$.mp.
(15) (population based or population-based).mp.
(16) (community based or community-based)mp.
(17) (whole of community or whole-of community).mp.
(18) (community wide or community-wide).mp.
(19) national.mp.
(20) state.mp.
(21) regio$.mp.
(22) local.mp.
(23) municip$.mp.
(24) district.mp.
(25) town$.mp.
(26) village$.mp.
(27) borough.mp.
(28) precinct.mp.
(29) (county or counties).mp.
(30) area.mp.
(31) province.mp.
(32) shire.mp.
(33) urban.mp.
(34) rural.mp.
(35) (city or cities).mp.
(37) (school based or school-based).mp.
(38) (secondary school or secondary-school).mp.
(39) (elementary school or elementary-school or primary school or primary-school).mp.
(40) (pre-school or preschool).mp.
(41) pupil$.mp.
(42) student$.mp.
(43) kindergarten$.mp.
(44) childcare.mp.
(45) nurser$.mp.
(46) daycare.mp.
(47) worksite$.mp.
(48) worksite$.mp.
(49) employee$.mp.
(50) worker$.mp.
(52) intervention study.mp.
(53) prevention.mp.
(54) primary prevention.mp.
(55) program$.mp.
(56) (community intervention$ or community-intervention$).mp.
(57) (community program$ or community-program$).mp.
(58) (health promotion or promotion).mp.
(59) (lifestyle intervention or life-style intervention).mp.
(60) exercise intervention.mp.
(61) (physical activity or physical actvity intervention).mp.
(62) (diet$ intervention or healthy eating intervention).mp.
(63) environment$ intervention.mp.
(64) policy.mp.
(65) policy implementation.mp.
(66) project.mp.
(67) study.ti.
(68) (randomi#ed control stud$ or randomi#ed control trial or RCT).mp.
(69) cohort stud$.mp.
(70) longitudinal.mp.
(71) prospective.mp.
(72) case control stud$.mp.
(73) case series.mp.
(74) (cluster-randomi#ed or cluster randomi#ed or randomi#ed).mp.
(75) quasi-experimental design.mp.
(76) interrupted time series.mp.
(77) pilot study.mp.
(78) program$ evaluation.mp.
(79) effectiveness.mp.
(80) evaluation.mp.
(81) (follow up or follow-up).mp.
(82) 1 or 2 or 3 or 4 or 5 or 6
(83) 7 or 8 or 9 or 10 or 11 or 12 or 13
(84) 14 or 15 or 16 or 17 or 18 or 19 or 20 or 21 or 22 or 23 or 24 or 25 or 26 or 27 or 28 or 29 or 30 or 31 or 32 or 33 or 34 or 35 or 36 or 37 or 38 or 39 or 40 or 41 or 42 or 43 or 44 or 45 or 46 or 47 or 48 or 49 or 50
(85) 51 or 52 or 53 or 54 or 55 or 56 or 57 or 58 or 59 or 60 or 62 or 64 or 65 or 66
(86) 67 or 68 or 69 or 70 or 71 or 72 or 73 or 74 or 75 or 76 or 77 or 78 or 79 or 80 or 81
(87) 82 and 83 and 84 and 85 and 86
(88) Limit to English

**Table 2:** Descriptive characteristics and assessment of setting-based interventions.

Author (Country) Study name	Study design Outcome Total follow-up	Participants	Gender as % of females Mean age (SD) Mean years of education (SD) or SESc	Number of participants, settings, or communities/randomisation units/response rate/baseline/lost to follow-up	Choice of setting/community	Intervention implemented	Changes in BMI as % or mean (*p* value or CI%)^e^
*Community-based*							
Jenum et al. [39] Jenum et al. [59]^a^ (Norway)	Quasiexperimental changes in PA, smoking, BMI, BP, lipids, and glucose 3 yrs. (cohort)	(I) Whole of community (30–67 yrs.) (C) Age-matched sample from neighbouring community	I: 57.2% C: 55.7% I: 47.7 yrs. (10.3) C: 48.0 yrs. (9.4) Mean years of education (SD): I: 11.6 yrs. (3.8) C: 12.2 yrs. (3.8)	(I) One district *n* = 6,700 (All 30–67 yrs. invited) (C) One neighbouring district Response rate: 48% Baseline: (I) *n* = 1497 (C) *n* = 1453 Lost to follow-up: (I) 41% (C) 40% No randomisation	(I) District selected as disadvantaged, with a high population of low-income, multiethnic residents (C) Neighbouring district selected due to demographic similarities	3-year intervention (same as follow-up) Individual: PA, diet, and smoking (a) Individual counselling: offered during biannual fitness tests and as per protocol for high-risk groups Environmental: PA (a) Awareness campaign: mass media, local meetings, pamphlets, and reminders to use stairs not lifts (b) Organised activities (free): walking groups, indoor activities, and tailored activities for students enrolled in language school (c) Infrastructure: improved safety of pavements and trails, including street lighting and labelling of walking trails (d) Worksite: encouragement of PA for staff employed at the local community organisation	Proportion with net increase in BMI (difference of proportion with increase and proportion with decrease) (I) 14.2% (C) 28.9% (*p* < 0.001)

Lupton et al. [45] (Norway) “Health inequalities in Finnmark programme-health and well-being project”	Quasiexperimental changes in PA, diet, smoking, BMI, BP, and cholesterol 6 yrs. (cohort)	(I) Whole of community (20–62 yrs.) (C) similar communities	I: 48.9% C: 49.8% Male: I: 47.7 yrs. (NA) C: 48.6 yrs. (NA) Female: I:47.5 yrs. (NA) C:47.9 yrs. (NA) Mean years of education (SD): Male I: 8.7 (NA) C: 8.5 (NA) Female I: 8.6 (NA) C: 8.4 (NA)	(I) One municipality *n* = 2500 (All 40–62 yrs. invited and 15% random sample of 20–39 yrs. invited) (C) Three municipalities *n* = 5000 (all 40–62 yrs. invited and 15% random sample of 20–39 yrs. invited) Response rate: NA Baseline: (I) *n* = 364 (C) *n* = 960 Lost to follow-up: (I) 39% (C) 30% No randomisation	(I) Municipality selected based on high CVD mortality in Finnmark county (C) Selected due to similarities in age, ethnicity, and main industry (fishing)	3-year intervention (shorter than follow-up) Individual: PA, diet, and smoking (a) Cholesterol screening: in food stores, provision of healthy recipes, and menus (b) Individual counselling: in primary care for persons at high risk identified though project's baseline screening Environmental: PA, diet, and smoking (a) awareness campaign: newspaper, radio, and TV (b) organised activities (tailored to specific groups or whole community, run by sports club or community originations): aerobics, physical training, badminton, swimming training, community dances, hiking tours, cooking classes, and health fair (c) Infrastructure: improved cycling paths and ski trails (d) Policy: smoke-free institutions and health care centers (e) Worksite: reestablishment of staff sporting association (sporting competitions) and provision of healthy vacuumed packed meals to fishermen (f) Supporting material: project manual	Mean BMI change Males (I) 1.50 (NA) (C) 1.1 (NA) (*p* < 0.002) Females (I) 1.9 (NA) (C) 1.4 (NA) (*p* < 0.001)
Kumpusalo et al. [42] Kumpusalo et al. [65]^a^ (Finland) “Finnish healthy village study” -A pilot study	Quasiexperimental changes in PA, diet, smoking, alcohol, BMI, BP, and lipids 3 yrs. (cohort + cross-sectional samples)	(I) Whole of community (20–64 yrs.) (C) Similar communities	I: 46.0% C: 46.4% 20–34 yrs. *n* = 245 35–49 yrs. *n* = 250 50–64 yrs. *n* = 298 NA by intervention Mean years of education (SD): NA Education level: (High^d^: 3%) NA by intervention	(I) Four villages (C) Two villages *n* = 220–490 Response rate: 80% Baseline: (I) *n* = 450 (C) *n* = 343 Lost to follow-up: (I) 11% (C) 21% No randomisation	Villages selected due to similar characteristics of rural villages associated with population, age, trades and services (I) Health profile available for two villages out of four (C) Health profile available for the two villages	3-year intervention (same as follow-up) Tailored components to each community Individual: PA (a) Walking tests (part of walking campaign) Environmental: diet, PA, and smoking (a) Awareness campaign: newspaper, radio, project booklet (b) Organised activities: (i) village seminars 1/month (e.g., diet, social support, and medicine), (ii) Study groups, sports groups, and courses e.g., healthy cooking, quitting smoking (iii) Walking campaigns 2/yr	Mean BMI before-after (I) 26.6 (4.7) versus 27.0 (4.7) (*p*=NS) (I) 26.1 (4.1) versus 26.4 (4.2) (*p*=NS) (C) 26.5 (4.1) versus 27.1 (4.0) (*p*=NS) (C) 26.9 (4.3) versus 27.4 (4.4) (*p*=NS) Test for comparisons between (I) and (C): NA

Isacsson et al. [37] (Sweden) “Halsan I Olofstrom” (HIO)	Pre- and post-intervention (No control group) changes in smoking, BMI, BP, cholesterol, and glucose 4 yrs. (cohort + cross-sectional samples)	(I) Whole of community (30–64 yrs. and children)	49.2% Male: 47.5 yrs. (9.6) Female: 47.5 yrs. (9.2) Mean years of education (SD): NA	One municipality *n* = 15,000 (All 30–64 invited and 30–64 who visited the health centre) Response rate: Survey 1: 79% Survey 2: 70% Survey 3: 74% Baseline: Survey 1: (1989) *n* = 347 Survey 2: (1991) *n* = 312 Survey 3: (1993) *n* = 325	Municipality selected based on high CVD mortality	5-year intervention (longer than follow-up) Individual: diet, PA, smoking and alcohol (a) Health screening for visitors to the health center, by invitation and at community activities. Pamphlets provided (b) Individual counselling in primary care for persons at high risk. Environmental: diet, PA, smoking, and alcohol (a) Capacity building in the community: school food service, teachers, health professionals, businesses, sporting clubs, worksites, restaurant employees, volunteers from NGO's (b) Awareness campaign: newspaper, radio, pamphlets, recipes, and project magazines delivered to households (c) Organised activities: Lectures by key persons in community and local area meetings promoting a healthy lifestyle and participation in local PA program to individuals and community groups (d) Food stores and restaurants: discount campaign for low fat, high fibre foods, and complementary recipes distributed (e) School: health education provided to school canteen managers and teachers One school received a health education program for children 7–13 yrs. and individual screening with GP (f) Worksites: larger worksites distributed health information and recipes to employees. Managers and union members were offered some health education	Mean BMI for every cross-sectional survey Males 1989: 25.9 (0.23) 1991: 26.3 (0.26) 1993: 26.4 (0.27) (*p*=NS) Females 1989: 25.3 (0.33) 1991: 25.4 (0.35) 1993: 25.1 (0.30) (*p*=NS)
Lingfors et al. [44] (Sweden) “Live for life”	Pre- and postintervention (no control group) changes in BMI, BP, and cholesterol 8 yrs. (cohort + cross-sectional samples)	(I) Whole of community (30 and 35 yrs.)	51.8% 30 yrs. *n* = 872 35 yrs. *n* = 1,637 Mean years of education (SD): NA	One county *n* = 272,215 (All 30 and 35 yrs. invited) Response rate: Year 1: NA Year 2: 72% Year 3: 63% Year 4: 67% Year 5: 55% Year 6: 52% Year 7: 60% Year 8: 58% Baseline: Year 1: *n* = 2509 Year 2: *n* = 3227 Year 3: *n* = 2878 Year 4: *n* = 2493 Year 5: *n* = 1884 Year 6: *n* = 1538 Year 7: *n* = 1545 Year 8: *n* = 1527	County selected based on high CVD mortality	8-year intervention (same as follow-up) Individual: diet, PA, smoking, alcohol, and stress (a) Health screening at health centers for those aged 30 and 35 yrs (b) Individual counselling provided to all screened participants. High-risk participants provided with additional support (individually or in groups) Environmental: diet and healthy lifestyle (a) Awareness campaign: radio and project newsletter (b) Food stores: education for staff and diploma program for stores meeting criteria for the promotion of healthy food	Mean BMI before-after for age groups 30 and 35 years Males Age 30 1989–1990: 24.8 (3.2) 1995–1996: 25.0 (3.2) (*p*=NS) Age 35 1989–1990: 24.8 (3.0) 1995–1996: 25.7 (3.4) (*p* < 0.001) Females Age 30 1989–1990: 23.3 (3.5) 1995–1996: 24.3 (4.4) (*p* < 0.001) Age 35 1989–1990: 23.6 (3.7) 1995–1996: 24.7 (4.1) (*p* < 0.001)
Weinehall et al. [56] Brannstrom et al. [57]^a^ (Sweden) “Norjso”	Quasiexperimental changes in smoking, BMI, BP, and cholesterol 4 yrs. (cohort + cross-sectional samples)	(I) Whole of community (30, 40, 50 and 60 yrs.) (C) Monica randomly selected, age-stratified reference population from same region Northern Sweden (25–64 yrs.)	I: 50.7% C: 49.3% I: 30 yrs. 21.7% 40 yrs. 26.2% 50 yrs. 23.0% 60 yrs. 29.2% C: 30 yrs. 22.3% 40 yrs. 25.7% 50 yrs. 26.9% 60 yrs. 25.1% Mean years of education (SD): NA Education level: High^d^ I: 17.9% C: 22.1%	(I) One municipality *n* = 5,500 (All 30, 40, 50, 60 yrs. invited) (C) One region *n* = 510,000 (*n* = 2,000 inhabitants 25–64 yrs. randomly selected) Response rate: (I) 1985: 96% (I) 1986: 96% (I) 1987: 96% (I) 1988: 96% (I) 1989: 94% (I) 1990: 91% (I) 1991: 88% (I) 1992: 81% (C)1986: 81% (C)1990: 79% Baseline: (I) 1985: *n* = 271 (I) 1986: *n* = 260 (I) 1987: *n* = 258 (I) 1988: *n* = 251 (I) 1989: *n* = 232 (I) 1990: *n* = 227 (I) 1991: *n* = 205 (I) 1992: *n* = 189 (C) 1986: *n* = 1625 (C) 1990: *n* = 1583 (The sample from 1986 was followed-up in 1988 and 1991) Lost to follow-up: 36% No randomisation	Municipality selected due to high CVD incidence and mortality	4-year intervention (same as follow-up) Individual: diet and healthy lifestyle (a) Health screening at health centers for age groups 30, 40, 50, and 60 yrs. annually (b) Individual counselling provided to all screened participants. High-risk participants provided with additional support Environmental: diet, PA, alcohol, and psychosocial factors (a) Awareness campaign: newspapers, radio, and TV (b) Organised activities: Educational and health promotion activities provided by existing community and sporting associations. Alternative methods using drama, music, and informal gatherings were encouraged (c) Food stores: food labelling system introduced	Mean BMI for every cross-sectional survey for (I) and (C) groups Males (I) 1985: 25.6 (NA) (I) 1986: 25.5 (NA) (I) 1987: 25.3 (NA) (I) 1988: 24.8 (NA) (I) 1989: 26.3 (NA) (I) 1990: 26.5 (NA) (I) 1991: 25.7 (NA) (I) 1992: 26.2 (NA) (*p* < 0.05) (C) 1986: 25.6 (NA) (C) 1990: 25.9 (NA) (*p*=NS) Females (I) 1985: 25.0 (NA) (I) 1986: 25.0 (NA) (I) 1987: 24.9 (NA) (I) 1988: 25.5 (NA) (I) 1989: 25.0 (NA) (I) 1990: 25.7 (NA) (I) 1991: 26.2 (NA) (I) 1992: 25.5 (NA) (*p*=NS) (C) 1986: 25.0 (NA) (C) 1990: 25.0 (NA) (*p*=NS) Mean BMI before-after Males (I) 25.4 (NA) versus 25.3 (NA) versus 25.1 (NA) (*p*=NS) Females (I) 25.0 (NA) versus 25.0 (NA) versus 25.1 (NA) (*p*=NS)
Schuit et al. [52] (The Netherlands) “Hartslag Limburg” (Heartbeat Limburg)	Quasiexperimental Changes in smoking, BMI, BP, lipids, and glucose 5 yrs. (cohort)	(I) Whole of community (20–59 yrs.) (C) Community originated from the same monitoring study as the intervention community	I: 49.6% C: 54.0% Male: I: 50.6 yrs. (9.8) C: 52.2 yrs. (9.9) Female: I: 50.6 yrs. (9.7) C: 51.3 yrs. (10.4) Mean years of education (SD): NA Low SES: Male: I: 45% C: 43% Female: I: 61% C: 61%	(I) One province *n* = 13,184 (*n* = 4,500 adult inhabitants randomly selected) (C) Same monitoring study (All 1,115 invited from ongoing cohort) Response rate: 80% Baseline: (I) *n* = 3,000 (C) *n* = 895 Lost to follow-up: (I) 19% (C) 15% No randomisation	Province selected as a demonstration project and from previous national monitoring studies	5-year Intervention (same as follow-up) 790 components implemented: PA, diet, and smoking: Individual: diet (a) Computer-tailored nutrition education Environmental: PA, diet, and smoking (a) Awareness campaign: radio, newspaper, TV, and pamphlets (b) Organised activities: establishment of walking/cycling clubs and associated campaign, stop-smoking campaign (c) Food stores: supermarket tours, food labelling (d) Policy: smoke-free areas	Mean BMI change Males (I) 0.37 (NA) (C) 0.71 (NA) (*p* < 0.05) Females (I) 0.38 (NA) (C) 0.63 (NA) (*p* < 0.05)

*Worksite-based*							
Engbers et al. [34] (The Netherlands) “Food steps”	Quasiexperimental, Changes in PA, diet, alcohol, smoking, BMI, BP, and lipids 1 yr. (cohort)	(I) Office workers from a governmental company with BMI > 23 kg/m^2^ (C) Office workers from a governmental company with BMI > 23 kg/m^2^	I: 37.4% C: 41.7% I: 45.3 yrs. (9.6) C: 45.5 yrs. (8.7) Mean years of education (SD): NA Education level: High^d^ I: 69.9% C: 63.9%	(I) One company located in one building (employees with BMI > 23 kg/m^2^ invited) (C) One company (different company) located in one building (employees with BMI > 23 kg/m^2^ invited) Response rate: 12% Baseline: (I) *n* = 257 (C) *n* = 283 Lost to follow-up: (I) 8% (C) 6% No randomisation	Worksites selected based on comparability of working environments	1-year Intervention (same as follow-up) Environmental: diet and PA (a) Awareness campaign: prompts (staircases and on elevator doors) to motivate and encourage stair use. Pamphlets promoting healthy lifestyles available in canteen (b) Canteen and vending machines: nutritional information provided at point of sale	Mean BMI change (I) −0.3 (1.2) (*p*=NS) (C) −0.2 (1.0) (*p*=NS) Test for comparisons between (I) and (C): NA

Kwak et al. [43] (The Netherlands) “NHF-NRG-in balance-project”	Quasiexperimental changes in body composition 2 yrs. (cohort)	(I) Blue collar and white collar workers employed by local government, hospital, factories, energy company, and university (<40 yrs.) (C) Blue collar and white collar workers (<40 yrs.) with a similar SES matched for similar SES	I: 50.7% C: 48.2% I: 38.9 yrs. (8.2) C: 35.0 yrs. (7.4) Mean years of education (SD): NA Education level: High^d^ I: 49.6% C: 51.6%	(I) Six worksites (Worksites that have accepted to be in the (I) group only, all employees <40 yrs. invited) (C) Six worksites (All employees <40 yrs. invited) Out of all 128 randomly selected worksites from the same region Response rate: NA Baseline: (I) *n* = 365 (C) *n* = 188 Lost to follow-up: (I) 30% (C) 23% No randomisation	Worksites matched for SES selected based on size (100 employees +) and staff access to a canteen	1-year intervention (shorter than follow-up) Individual: diet and PA (a) Professional monitoring of body composition (b) “In-balance-box” (pedometer, measuring tape, “calorie guide,” food, and exercise diary) (c) Computer-tailored advice. Environmental: diet and PA Free choice of interventions by worksite including: (a) Awareness campaign: promotional material to encourage stair-use, lunch-walking, and cycling or an information wall about energy balance (b) Organised activities: health workshops (c) Canteen: healthy choices	Mean BMI change (I) −0.11 (1.4) (C) 0.03 (1.0) (*p*=NS)

Hedberg et al. [36] (Sweden)	Quasiexperimental changes in PA, diet, smoking, BMI, BP, lipids and stress 1½ yrs. (cohort)	(I) Professional drivers, 86% blue collar workers (C) Professional drivers, 92% blue collar workers	No females I: 42.9 yrs. (9.9) C: 43.4 yrs. (10.6) Mean years of education (SD): NA	(I) Drivers within 50 km from one town (51 drivers invited) (C) Drivers within 50 km from another town (51 drivers invited) Response rate: 95% Baseline: (I) *n* = 49 (C) *n* = 48 Lost to follow-up: (I) 16% (C) 2% No randomisation	Participants selected from previous participation in a CVD screening program of professional drivers The 102 invited drivers did not differ compared to the other 260 drivers of the previous screening programme	1-year intervention (shorter than follow-up) Individual: diet, PA, smoking, and stress (a) Health and nutrition screening for all participants (b) Counselling for all participants led by a healthcare consultant and Dietitian. Pamphlets and free activities provided about a healthy lifestyle at the individual and group level Environmental: diet and PA (c) Organised activities: practical education sessions for drivers and their families. e.g., an exercise session and cooking classes (healthy lunch boxes)	Mean BMI before-after (I) 24.4 (NA) versus 24.9 (NA) (*p*=0.009) (C) 25.4 (NA) versus 25.8 (NA) (*p*=NS)
*School-based*							
Ask et al. [24] (Norway)	Quasiexperimental (pilot study) changes in diet and BMI 4 months (cohort)	(I) 10th grade students (15 yrs.) from a secondary school (C) 10th grade students (15 yrs.) from the same school	I: 42.3% C: 50.0% No mean age^b^ Mean years of education (SD): NA	(I) One school, one class *n* = 26 (C) Same school, one class *n* = 28 (Intervention school randomly selected among 2 schools) Response rate: 100% Baseline: (I) *n* = 26 (C) *n* = 28 Lost to follow-up: (I) NA (C) NA No randomisation	School selected due to request from teachers concerned about antisocial behaviour and poor attendance	4-month intervention (same as follow-up) Environmental: diet (a) Food provision: free healthy breakfast served to students each school day	Median (range) BMI before and after Males (I) 22.6 (17.8–33.6) versus 21.8 (17.6–33.9) (*p*=NS) (C) 21.7 (17.0–29.4) versus 22.4 (18.6–29.2) (*p* < 0.05) Females (I) 21.8 (16.9–27.3) versus 22.1 (17.5–28.1) (*p*=NS) (C) 21.6 (16.7–28.4) versus 22.1 (16.9–28.7) (*p* < 0.05) Test for comparisons between (I) and (C): NA

Ask et al. [25] (Norway)	Quasiexperimental (pilot study) changes in diet and BMI 4 months (cohort)	(I) 9th grade students from a secondary school (C) 9th grade students from secondary schools in the same region	NA No mean age Mean years of education (SD): NA	(I) One school *n* = 64 (C) Two schools *n* = 120 (Intervention school randomly selected among 3 schools) Response rate: 82% Baseline: (I) *n* = 61 (C) *n* = 95 Lost to follow-up: (I) 9% (C) 4% No randomisation	Schools selected as their syllabus for the 9th grade included lunch preparation in the home economics class, provided 3 times per week	4-month intervention (same as follow-up) Environmental: diet (a) Food provision: free healthy school lunch served to students (prepared by students and served in classroom)	Mean BMI before-after Males (I) 20.7 (3.1) versus 21.3 (3.3) (*p* < 0.001) (C) 20.8 (2.9) versus 21.2 (3.1) (*p* < 0.001) (*p*=NS) Females (I) 20.5 (3.5) versus 20.7 (3.4) (*p*=NS) (C) 20.2 (2.8) versus 20.5 (2.5) (*p* < 0.05) (*p*=NS)
Bere et al. 2014 [26] (Norway) “Norwegian school fruit program for free”	Cluster randomised changes in diet and BMI 8 yrs. (cohort)	(I) 6th and 7th grade children (10–12 yrs.) from schools from one county (C) 6th and 7th grade children (10–12 yrs.) from schools in the same and an alternative county	I: 49% C: 50% 11.8 yrs. (NA) NA by intervention Parents with a high education: I: 48% C: 39%	(I) Nine schools *n* = NA (C) Twenty-nine schools *n* = NA (38 elementary schools, randomly selected from two counties) Response rate: NA Baseline: (I) *n* = 585 (C) *n* = 1365 Lost to follow-up: (I) 81% (C) 85% Randomisation	(I) Schools selected from one county participating in the “fruit and vegetables make the marks project” (FVMM) (C) Schools selected from 2 counties participating in the same project	1-year intervention (shorter than follow-up) Environmental: diet (a) Food provision: free fruit for students at school (one piece of extra fruit per day)	Mean BMI (95% CI) (I) NA versus 20.5 (19.9, 21.1) versus 22.7 (22.0, 23.4) (C) NA versus 20.7 (20.2, 21.3) versus 23.2 (22.6, 23.8) (*p*=NS)

Grydeland et al. [35] (Norway) “Health in adolescents (HEIA) study”	Cluster Randomised changes in body composition 20-months (cohort)	(I) 6th grade children (11 yrs.) from schools in the largest towns/ municipalities of 7 counties (C) 6th grade children (11 yrs.) from schools from the same region	I: 50% C: 48% No mean age Parents with an education >16 yrs. I: 36.3% C: 31.1%	(I) Twelve schools *n* = 784 (C) Twenty-five schools *n* = 1381 (Out of all 177 schools invited, 37 schools accepted) Response rate: 73% Baseline: (I) *n* = 566 (C) *n* = 1014 Lost to follow-up: (I) 7% (C) 9% Randomisation	Schools selected from large municipalities located in 7 counties from the same region with greater than 40 students in 6th grade	20-month intervention (same as follow-up) Individual: diet and PA (a) Computer-tailored individual advice Environmental: diet and PA (a) Curriculum: e.g., Lessons on diet and PA (1/month), breaks for PA and fruit and vegetable snacks (1/week), and active transport campaigns (b) Teachers: training for PE teachers to increase active involvement and enjoyments of students in PE. Toolbox for teachers: Student workbooks, sports equipment, pedometers, practical nutrition activities, and box of sports equipment provided for children to access during breaks (c) Parents: pamphlets on healthy lifestyle (monthly) School-wide: annual meetings for staff and parent committee to encourage active participation and support of project, and positive environmental changes within the school grounds	Mean BMI change (95% CI) Males (I) NA versus 18.6 (18.5; 19.3) (C) NA versus 18.5 (18.4; 18.6) (*p*=NS) Females (I) NA versus 19.0 (18.8; 19.3) (C) NA versus 19.2 (19.1; 19.3) (*p*=significant; NA);
Resaland et al. [51] (Norway) “The sogndal school-intervention study”	Quasiexperimental changes in BMI, BP, lipids, and glucose 2 yrs. (cohort)	(I) 4th grade children (9 yrs.) from a school in a municipality (C) 4th grade children (9 yrs.) from a school from another municipality with similar SES	I: 49.6% C: 52.7% No mean age Mean years of education (SD) NA	(I) One school *n* = 125 (C) One school *n* = 134 Response rate: 99% Baseline: (I) *n* = 125 (C) *n* = 131 Lost to follow-up: (I) 26% (C) 37% No Randomisation	Schools selected from municipalities located within the same region, 105 km apart and had a similar SES, similar size, and similar number of children	2-year intervention (same as follow-up) Environmental: PA (a) Curriculum: 60 minutes of PA per day of the school week (includes 90 minutes per week of standard school-based PE)	Mean BMI change (I) 0.8 (0.1) (C) 0.9 (0.1) (*p*=NS)

Bugge et al. [27] (Denmark) “Copenhagen school child intervention study” (CoScIS)	Quasiexperimental Changes in PA, BMI, BP, lipids, and glucose 7 yrs. (cohort)	(I) 1st-3rd grade children (6-7 yrs.) from schools from one local authority (suburb) (C) 1st-3rd grade children (6-7 yrs.) from schools from another local authority with similar SES	I: 45.6% C: 50.3% Male: I: 6.8 yrs. (0.4) C: 6.8 yrs. (0.3) Female: I: 6.7 yrs. (0.4) C: 6.6 yrs. (0.4) Mean years of education (SD): NA	(I) Ten schools *n* = NA (C) Eight school *n* = NA Response rate: 69% Baseline: (I) *n* = 408 (C) *n* = 286 Lost to follow-up: (I) 36% (C) 37% No Randomisation	(I) Schools selected due to an interest by one of the local authorities, in measuring the effect of recently upgraded PA opportunities for young school children (C) Schools selected due to similar SES	3-year intervention (shorter than follow-up) Environmental: PA and diet (a) Curriculum: 180 minutes of PE per week (includes 90 minutes per week of standard school-based PA). Theoretical lessons on PA and healthy eating (b) Teachers: training for PE teachers in use of specialised didactic tools to motivate children to participate and enjoy PA (c) Infrastructure: upgrade of school sports and playing facilities	Mean BMI change Baseline to T1 (I) 1.31 (1.23) (C) 1.15 (1.20) (*p*=NS) Baseline to T2 (I) 3.40 (1.94) (C) 3.07 (1.78) (*p*=0.057)

Klakk et al. [40] (Denmark) “CHAMPS study-DK”	Quasiexperimental changes in body composition 2 yrs. (cohort)	(I) 2nd–4th grade children (8–13 yrs.) from schools within one municipality (C) 2nd–4th grade children (8–13 yrs.) from schools within the same municipality with similar SES	NA	(I) Six schools *n* = 773 (C) Four schools *n* = 734 (Out of all 19 invited for (I) and out of all 6 invited for (C)) Response rate: 80% Baseline: (I) 402 (C) 315 Lost to follow-up: (I) 8.2% (C) 8.0% No randomisation	(I) Schools selected based on an initiative by a community to increase PE lessons in local primary schools for improved health of students (C) Schools matched by SES, school size, and rural/urban area	2-year intervention (same as follow-up) Environmental: PA (a) Curriculum: minimum of 4.5 hours of PE per week (includes 90 minutes per week of standard school-based PE) (b) Teachers: training of PE teachers to plan and facilitate age-related PA for children	Mean BMI before-after (I) 16.7 (2.2) versus 17.7 (2.5) (C) 16.8 (2.1) versus 17.9 (2.6) (*p*=NS)
Puska et al. [50] (Finland) “The North Karelia youth project”	Quasiexperimental changes in diet, smoking, BMI, BP, cholesterol, health knowledge, attitude, and emotional problems 2 yrs. (cohort)	(II)^d^ 7th grade students (13 yrs.) from schools from one county (CI)^d^ 7th grade students (13 yrs.) from schools from the same county (C) 7th grade students (13 yrs.) from schools from another county	II: 44.8% CI: 47.6% C: 51.8% No mean age Mean years of education (SD): NA	(II) Two schools *n* = 338 (CI) Two schools *n* = 318 (C) Two schools *n* = 310 (One of the two major schools from the county capital randomly selected and one of the schools of major rural centers randomly selected.) Response rate: 99% Baseline: (II) *n* = 335 (CI) *n* = 315 (C) *n* = 309 Lost to follow-up: (II) 12% (CI) 10% (C) 11% Randomisation	(II) (CI) The Intervention county (North Karelia) was selected as it was the setting of an established ‘whole of community' intervention, of which this school-based intervention was a component (C) County selected as it was located in the same regional area as the (I) county	2-year intervention (same as follow-up) Part of a whole of community intervention (II) Intensive intervention in 2 schools implemented by the project team Individual: diet, PA, and smoking (a) Health screening: by school nurse 1-2/yr.) Counselling: a health passport was used to guide lifestyle counselling for the children by the school nurse. Additional in-home consultations provided by a nutritionist for those children at high risk of CVD Environmental: diet, PA, and smoking (a) Awareness campaign: to promote lifestyle changes (mass media, project magazine, posters, and pamphlets) (b) Curriculum: antismoking (10 × 45 minutes sessions over 2 yrs., led by trained older peer leaders) and diet (education sessions about healthy eating) (c) Teachers: active participation in project encouraged (d) Parents: education sessions promoting a healthy lifestyle (e) Canteen: nutritional changes to the lunch provided to include less total and saturated fat, higher proportion of polyunsaturated fat, more fibre, and less sodium (CI) County-wide Intervention in remainder of North Karelia (i) Recommendations and training regarding the interventions applied in the (II) schools was given to the (CI) schools. Implementation of these initiatives by the schools was encouraged	Mean BMI change Males (II) 1.4 (1.3) (CI) 1.3 (1.3) (C) 1.5 (1.0) (*p*=NS) Females (II) 1.4 (1.2) (CI) 1.2 (1.4) (C) 1.4 (1.2) (*p*=NS)
Magnusson et al. [46] (Iceland)	Cluster Randomised changes in body composition and cardiorespiratory fitness 2 yrs. (cohort)	(I) 2nd grade children (7 yrs.) from schools from the same city (C) 2nd grade children (7 yrs.) from schools from the same city	I: 50.8% C: 60.1% No mean age Mothers with a university degree: I: 52.1% C: 62.9% Fathers with a university degree: I: 43.9% C: 46.2%	(I) Three schools *n* = 151 (C) Three schools *n* = 170 (One school from each pair was randomised to (I) and the other to (C)) Response rate: 83% Baseline: (I) *n* = 128 (C) *n* = 138 Lost to follow-up: (I) 20% (C) 41% Randomisation	(I) Schools in this region were selected based on a national concern of a decline in aerobic fitness of children and adolescents (C) Schools matched for school size and grades	2-year intervention (same as follow-up) Environmental: PA and diet (a) Curriculum: increase of PA at school through playful learning and participation of teachers together with students in activities e.g., outdoor teaching, excursions, promotion of active transport, one additional PE lesson per week to represent 3 × 40 minute sessions per week. (includes 2 × 40 minute sessions per week of standard school-based PE). Nutrition education lessons to improve awareness, knowledge, self-efficacy, taste and preference surrounding healthy eating with the aim of increasing fruit and vegetable intake at school and home (b) Teachers: training of general teachers to improve their health promoting skills at bimonthly meetings with research team. Education Toolbox provided (books, DVD's, and sporting and play equipment for indoor and outdoor use) (c) Parents: achieving positive parental influence towards healthy eating an aim of the intervention	Mean BMI before-after (I) 16.0 (1.8) versus 17.4 (2.2) (C) 16.7 (2.1) versus 17.5 (2.7) (*p*=NS)
Elinder et al. [33] Elinder et al. [66]^a^ (Sweden) “Stockholm county implementation programme- SCIP”	Quasiexperimental changes in PA, diet, BMI, and self-esteem 2 yrs. (cohort)	(I) 2nd, 4th, and 7th grade children and students (6–16 yrs.) from schools in a municipality (C) 2nd, 4th, and 7th grade children and students (6–16 yrs.) from schools from the same municipality	Grade 2: 49.2% Grade 4: 52.3% Grade 7: 47.6% Grade 2: Male: 8.8 yrs. (0.02) Female: 8.7 yrs. (0.03) Grade 4: Male: 10.8 yrs. (0.02) Female: 10.8 yrs. (0.03) Grade 7: Male: 13.9 yrs. (0.03) Female: 13.9 yrs. (0.03) NA by intervention Parents with a high education (>12 years at follow-up): Grade 2: Male: 65.5% Female: 69.6% Grade 4: Male: 62.0% Female: 65.0% Grade 7: Male: 60.2% Female: 56.1%	(I) Nine schools *n* = 764 (C) Nine schools *n* = 595 (Self-selection to (I) or (C) group out of all the 18 invited) Response rate: 60% Baseline: (I) *n* = 482 (C) *n* = 331 Lost to follow-up: (I) 6% (C) 13% No Randomisation	(I) Schools located in a middle-class municipality were selected for the study due to a request by representatives from the municipality (C) Schools from the same municipality who did not accept to participate in the intervention (Project part of the Stockholm County Overweight and Obesity Action plan)	2-year intervention (same as follow-up) Environmental: diet, PA, and mental health (a) Awareness campaign: newsletters, pamphlets (a) Teachers: 4 training sessions in health promotion, diet, PA, and mental health. Education toolbox provided (including written health education material) (b) Parents: minimum of 1 meeting with parents conducted by the school and research team where the project was presented and pamphlets on health information provided (c) School-wide: each school, in collaboration with a multidisciplinary health team, through a series of workshops developed a tailored action plan with 4 themes (health practices, PA, mental health, and diet) Example of implemented strategies from a combination of various schools: curriculum: outdoor activities, activities on body image, and encouragement of students to prepare healthy snacks Teachers: training skills associated with empathy Parents: encouraged to provide a healthy breakfast and initiate active transport e.g., walking school bus Infrastructure: improvement to playground Policy development/guidelines: implementation of school guidelines to reduce sweets served at festivities	(*p*=NS)
Marcus et al. [47] (Sweden) “STOPP”	Cluster randomised changes in PA, diet, and BMI 4 yrs. (cohort)	(I) Children (6–10 yrs.) from schools in one county area (C) Children (6–10 yrs.) from schools, from the same county area	49% NA by intervention I: 7.4 yrs. (1.3) C: 7.5 yrs. (1.3) Parents with an education higher than upper secondary school: I: 23–46% C: 26–46%	(I) Five schools *n* = NA (C) Five schools *n* = NA (Out of 387 invited schools, 170 schools accepted and 10 schools were selected) Response rate: 90–100% Baseline: (I) *n* = 1670 (C) *n* = 1465 Lost to follow-up: 89% Randomisation	Selected schools had a population of students from families of middle and working class	4-year intervention (same as follow-up) School and after School care Environmental: diet and PA (a) Awareness campaign: newsletter for parents and school staff biannually (b) Curriculum: 30 min extra PA per day by general teachers (c) Parents: encouraged not to provide students with unhealthy food and drinks at school or for school outings (d) Canteen: improvements made to the standard free school lunch menu to include less fat, sugar, and more fibre with the promotion of fruit and vegetables (e) Policy implementation/guidelines (i) Restricted of access to and time spent playing computer games to 30 min per school day (ii) Reduced use of sweetened foods at birthday parties and excursions	NA

Nyberg et al. [49] (Sweden) “The healthy school start study”	Cluster randomised changes in PA, diet, BMI, health behaviours, and parental self-efficacy 1 yr. (cohort)	(I) Children (6 yrs.) and their parents from preschools in a municipality (C) Children (6 yrs.) and their parents from preschools within the same municipality	I: 47.3% C: 50.9% No mean age Parents with a low education: I: 33% C: 40%	(I) Seven preschool classes *n* = NA (C) Seven preschool classes *n* = NA *N* total = 338 (Out of all 15 eligible schools in the area, 8 schools accepted and included 14 preschool classes) Response rate: 72% Baseline: (I) *n* = 129 (C) *n* = 112 Lost to follow-up: (I) 2% (C) 0% Randomisation	Schools selected from a municipality with low to medium SES due to the higher prevalence of obesity in lower SES communities in Sweden	6-month intervention (shorter than follow-up) Environmental: diet and PA (a) Curriculum: 30-minute healthy lifestyle education sessions, held 7–10 times/intervention period. Toolbox of activities provided (teacher manual and student workbooks) (b) Teachers: 2-hour training provided for classroom activities (c) Parents: pamphlets provided (healthy eating, PA, screen time, and sleep), motivational interviews (2 × 45 minute sessions), active participation encouraged with children's healthy lifestyle homework	NA
De Henauw et al. [31] Hense et al. [67]^a^ Ahrens et al. 2011 [68]^a^ (Sweden) “IDEFICS” “The Identification and prevention of dietary- and lifestyle-induced health Effects in children and infants approach”	Quasiexperimental changes in diet, body composition, well being, screen time, and sleep 2 yrs. (cohort)	(I) Children (2–9.9 yrs.) from kindergartens and primary schools from one region (C) Children (2–9.9 yrs.) from kindergartens, preschools, and schools (grades 1 and 2) from a region with similar SES	48.8% NA by intervention 5.7 yrs. (0.05) NA by intervention Parents with a high education: 67.2% NA by intervention	All the schools in the region were invited Number of randomisation units NA *N* total = 2759 Response rate: 66% Baseline: (I) *n* = 902 (C) *n* = 907 Lost to follow-up: 18.2% No randomisation	(I) Community selected as one of eight European countries as part of the IDEFICS cross-cultural childhood obesity and prevention study (C) Community selected based on similar size and SES	2-year intervention (same as follow-up) Environmental: diet, PA, stress, and sleep (a) Awareness campaign: local media to promote a healthy lifestyle in the community (b) Curriculum: increased opportunities for PA and provision of healthy lifestyle education (c) Parents: encouraged to support a healthy lifestyle for their children (d) Infrastructure (community): liaison with local authorities to improve e.g., outdoor play and cycling opportunities, access to water fountains (e) School-wide: improvement to the school food environment (f) Supporting materials: toolbox detailing implementation of intervention components focused on diet, PA, stress-coping capacity, and sleep quality	Mean BMI-z score before-after Males (I) 0.070 versus 0.138 (C) −0.127 versus −0.021 (*p*=NS) Females (I) 0.007 versus 0.104 (C) −0.093 versus −0.017 (*p*=NS)

Sollerhed and Ejlertsson 2008 [54] (Sweden)	Quasiexperimental Changes in PA and BMI 3 yrs. (cohort)	(I) Children (6–9 yrs.) from a school from a rural location (C) Children (6–9 yrs.) from a school from a rural location with similar SES	I: 39.7% C: 48.6% Mean age (SD): NA Mean years of education (SD): NA	(I) One school *n* = NA (C) One school *n* = NA *N* total = 132 Response rate: 100% Baseline: (I) *n* = 58 (C) *n* = 74 Lost to follow-up: 8% No randomisation	Schools selected based on similarities of rural location, size, appearance, structure, and SES of the children	3-year intervention (same as follow-up) Environmental: PA (a) Curriculum: increase in PE time to include one 40-minute lesson per day–4 days per week (includes standard school-based PE of one lesson/week (6–9 yrs.) and two lessons/week (10–12 yrs.) +60 minutes of outdoor activities with classroom teacher once per week Obese children offered one extra lesson per week	Mean BMI change (I) −0.32 (1.44) (C) 0.25 (1.58) (*p*=0.03)

Stenevi-Lundgren et al. [55] (Sweden) “Malmö pediatric osteoporosis prevention (POP) study”	Quasiexperimental changes in PA and body composition (I) 1 yr. (C) 2 yrs. (cohort)	(I) 1st and 2nd grade girls (7–9 yrs.) from a school from a middle-class area in a municipality (C) 1st and 2nd grade girls (7–9 yrs.) from neighbouring schools with similar SES	I: 100% C: 100% I: 7.7 yrs. (0.6) C: 7.9 yrs. (0.6) Mean years of education (SD): NA	(I) One school *n* = 61 (C) Three schools *n* = NA Response rate: (I) 90% (C) NA Baseline: (I) *n* = 55 (C) *n* = 64 Lost to follow-up: (I) 4% (C) 22% No randomisation	(I) School selected that did not have a high level of PA in the curriculum (C) Schools selected from neighbouring area with similar SES	1-year intervention (same as follow-up) Environmental: PA (a) Curriculum: One 40-minute lesson of PE per school day (200 min/week, includes standard school-based PE of 60 minutes PE/week)	Mean annual BMI change (95% CI) (I) 0.5 (0.2; 0.8) (C) 0.4 (0.2; 0.5) (*p*=NS)
Busch et al. [28] (The Netherlands) “The utrecht healthy school program (UHS)”	Quasiexperimental Changes in PA, diet, alcohol, drug use, smoking, BMI, sedentary time, sexual behaviours, and bullying 2 yrs. (cohort + cross-sectional samples)	(I) Students from high schools from suburbs of middle-large cities (C) Students from high schools, from suburbs of middle-large cities	NA	(I) Two schools *n* = 1400 (C) Two schools *n* = 1400 Response rate: 80% Baseline: 2011: *n* = 1716 2012: *n* = 1692 2013: *n* = 2393 Lost to follow-up: 65% No randomisation	(I) Schools selected to implement the Utrecht Health School (UHS) program (C) Schools selected from suburbs of middle-large cities as for the (I) group	2-year intervention (same as follow-up) Environmental: diet, PA, and smoking Priorities of (I) school A: increased PA, reduced sedentary time, healthy weight, nutrition, preventing, and reducing smoking Priorities of (I) school B: nutrition and PA Strategies implemented via capacity building through a tailored whole-school approach: (a) Capacity building: integration of local health authority e.g., professional support and provision of a Health Promoting Schools (HPS) coordinator (b) Curriculum: development of personal skills in health education. Health promoting schools goals guided the curriculum(c) Teachers: some unstructured competency training in health education provided (d) Parents: active involvement of parents to promote a healthy lifestyle (e) Canteen: healthy options provided (f) Policy: e.g., no smoking on school grounds Note: strategies implemented in a higher degree in (I) school A than (I) school B	School A Baseline versus T1 (*p* < 0.05) Baseline versus T2 (*p* < 0.05) School B Baseline versus T1 (*p*=NS) Baseline versus T2 (*p*=NS)
Busch et al. [29] (The Netherlands) “The utrecht healthy school (UHS) program”-Pilot	Pre- and Postintervention historical control group (pilot study for Busch et al.) [28] Changes in PA, diet, alcohol, drug use, smoking, BMI, sedentary time, sexual behaviours and bullying 3 yrs. (cross-sectional samples)	(I) 4th grade students (15–16 yrs.) from a secondary school (C) 4th grade students (15–16 yrs.) from the same school, enrolled 3 years earlier	I: 47% C: 54% Mean age (SD): NA Mean years of education (SD): NA	(I) One school *n* = 199 (C) Same school *n* = 220 (The (C) group came from 4th grade students in 2007) (3 yrs. before the 4th grade students in 2010) Response rate: (I) 60% (C) 100% Baseline: (I) *n* = 136 (C) *n* = 220	(I) School selected to implement the Utrecht Health School (UHS) program in 4th graders in 2010 (C) Students selected who were 4th graders in 2007 at the same school	3-year intervention (same as follow-up) Environmental: diet, PA, alcohol, smoking, drug use, sexual behaviour, bullying, sedentary activity, and excessive gaming/internet use 1st year priorities: nutrition, reducing alcohol, smoking, sedentary behaviours, and bullying 2nd year approach: PA, sexual behaviours, and reducing drug use Strategies implemented via a capacity-building through a tailored whole-school approach: (a) Capacity building: integration of local health authority e.g., professional support (b) Curriculum: innovative and interactive methods to develop personal skills e.g., handling peer pressure, with special teaching modules using peer education. Health Promoting Schools goals guided curriculum for each priority area (c) Teachers: in-service training by health professionals (d) Parents: active involvement of parents to promote a healthy lifestyle (e) Canteen: healthy options provided (f) Policy: no smoking, alcohol or drugs. Bullying-zero tolerance (g) Supporting materials: healthy school website created by the school	NA
de Greeff et al. [30] (The Netherlands) Part of the project “Fit en vaardig op school” (fit and academically proficient at school; F&V)”	Cluster randomised changes in BMI and fitness 22-weeks (cohort)	(I) 2nd or 3rd grade children (7–8 yrs.) from schools in one region (C) 2nd or 3rd grade children (7–8 yrs.) from schools from the same region	I: 55.2% C: 59.0% I: 8.0 yrs. (0.7) C: 8.2 yrs. (0.8) Mean years of education (SD): NA	(I) Six 2nd grade and six 3rd grade classes *n* = NA (C) Six 2nd grade and six 3rd grade classes *n* = NA *N* total = 388 (Out of 12 schools 2nd or 3rd grade class was randomised as (I) or (C) for each of the 12 schools) Response rate: 97% Baseline: (I) *n* = 181 (C) *n* = 195 Lost to follow-up: NA Randomisation	Schools were selected as they were part of the project “Fit en Vaardig op school,” a randomised trial with the aim to improve academic performance	22-week intervention (same as follow-up) Environmental: PA (a) Curriculum: integration of physically active academic lessons of 30 minute lessons, 3 times per week implemented by trained substitutes teachers	Mean BMI before-after 2nd grade (I) 16.4 (NA) versus 16.7 (NA) (*p*=NS) (C) 16.4 (NA) versus 16.6 (NA) (*p*=NS) 3rd grade (I) 17.0 (NA) versus 17.2 (NA) (*p*=NS) (C) 17.0 (NA) versus 17.6 (NA) (*p*=significant; NA) Test for comparisons between (I) and (C): NA

Kocken et al. [41] (The Netherlands) “Extra fit!” (EF!)	Cluster randomised changes in PA, diet, BMI, sedentary behaviour, and behavioural determinants 2 yrs. (cohort)	(I) 4th–6th grade children (9–11 yrs.) from schools (C) 4th–6th grade children (9–11 yrs.) from schools	I: 52.0% C: 51.3% I: 9.2 yrs. (0.6) C: 9.1 yrs. (0.6) Mean years of education (SD): NA	(I) Twenty-three schools *n* = NA (C) Twenty-two schools *n* = NA (Out of 500 schools from the same country, 65 were randomised, 20 dropped-out after randomisation. For every pair of school, one was randomised to (I) and the other to (C).) Response rate: NA Baseline: (I) *n* = 615 (C) *n* = 497 Lost to follow-up: (I) 40% (17 schools) (C) 5% (21 schools) Randomisation	(I) Children aged 9–11 yrs. were selected due to their ability to participate in the study questionnaires and the restricted budget for the study (C) Schools matched, based on similar SES and urbanization	2-year intervention (same as follow-up) Environmental: diet and PA (a) Curriculum: practical and interactive theoretical education program promoting behavioural changes towards a healthy diet and PA. Children participated in an average of 7.6 hours of lessons over 16-weeks per school year compared to control schools with an average of 3.3 hours. Schools could offer extra PA lessons at their discretion (b) Teachers: professional support provided to teachers by local health professionals and sports service (c) Parents: encouraged to promote a healthy lifestyle and participate with homework activities. Extra optional activity: “Extra fit-day” for parents and children	Mean BMI z-score before-after (I) 0.6 (0.2) versus 0.6 (1.2) versus 0.6 (1.1) (C) 0.6 (1.1) versus 0.5 (1.2) versus 0.6 (1.2) (*p*=NS)
de Meij et al. [32] (The Netherlands) “JUMP-in study”	Quasiexperimental Changes in PA, BMI, sports participation, and fitness 20-months (cohort)	(I) 3rd–8th grade children (6–12 yrs.) from schools in 2 city districts (C) 3rd–8th grade children (6–12 yrs.) from comparable schools in geographically separated city districts	I: 51.2% C: 48.1% Male: I: 8.6 yrs. (1.9) C: 8.6 yrs. (1.8) Female: I: 8.5 yrs. (1.9) C: 8.5 yrs. (1.8) Mean years of education (SD): NA	(I) Nine schools *n* = NA (C) Ten schools *n* = NA Response rate: 100% Baseline: (I) *n* = 1378 (C) *n* = 1451 Lost to follow-up: (I) 20% (C) 13% No randomisation	Schools were selected from socially and economic deprived areas which met the criteria of a certified PE teacher, high enrolment of students with a low SES, and access by school to a gymnasium	2-year intervention (same as follow-up) Individual: PA (a) Health screening for children-annually: “pupil follow-up system.” (b) Additional tailored activities for overweight children: “club extra” Environmental: PA (a) Curriculum: regular PA breaks in class time “The class moves.” In class activity workbook promoting PA, associated skills, and health benefits (b) Parents: workbook “this is your way to move” with activities for children and parents. Parental information services about sports activities, meetings, and courses (c) School-wide: different sporting activities offered to children to try on a daily basis in collaboration with local sports clubs “School sports clubs.”	Mean BMI before-after (I) 18.2 (3.4) versus 18.7 (3.6) versus 19.1 (3.7) (C) 18.1 (3.4) versus 18.4 (3.5) versus 18.8 (3.7) (*p*=NS)

Jansen et al. [38] (The Netherlands) “Lekker fit!” (enjoy being fit!)	Cluster randomised changes in BMI and fitness 2 yrs. (cohort)	(I) 3rd–8th grade children (6–12 yrs.) from schools from an inner-city area (C) 3rd–8th grade children (6–12 yrs.) from schools from the same inner-city area	Grades 3–5 I: 50.5% C: 51.0% Grades 6–8 I: 52.8% C: 49.0% Grades 3–5 I: 7.7 yrs. (1.0) C: 7.8 yrs. (1.0) Grades 6–8 I:10.8 yrs. (1.0) C:10.8 yrs. (1.0) Mean years of education (SD): NA	(I) Ten schools *n* = 1271 (C) Ten schools *n* = 1499 (Out of 27 schools that volunteered to participate, 26 were paired (one did not match) and randomised to (I) or (C)) Response rate: 95% Baseline: (I) *n* = 1240 (C) *n* = 1382 Lost to follow-up: (I) 7% (C) 8% Randomisation	Primary schools were selected as located in deprived inner-city neighbourhoods, with low SES, and a high proportion of immigrant children	2-year intervention (same as follow-up) Individual: diet and PA (a) Eurofit test with scorecard at commencement and conclusion of school year (b) Individual counselling by school nurse as required Environmental: diet and PA (a) Curriculum: 3xPE sessions per week (includes 2 PE sessions per week of standard school-based PE) Educational program on healthy lifestyle, diet, and PA (b) Parents: annual information meeting about local sporting clubs (c) Community: optional extra sport and play activities outside school hours in collaboration with local sporting clubs	Mean BMI before-after Grades 3–5 (I) 17.1 (2.8) versus 17.5 (3.0) (C) 17.1 (2.8) versus 17.6 (3.1) (*p*=NS) Grades 6–8 (I) 19.6 (4.0) versus 20.4 (4.2) (C) 19.1 (3.8) versus 19.8 (4.1) (*p*=NS)
Singh et al. [53] (The Netherlands) “Dutch obesity intervention in teenagers (DOiT)”	Cluster randomised changes in PA, diet and body composition 20-months (cohort)	(I) 1st grade students (12–14 yrs.) from schools (C) 1st grade students (12–14 yrs.) from schools	I: 53.2% C: 46.6% Males I: 12.8 yrs. (0.5) C: 12.9 yrs. (0.5) Females I: 12.6 yrs. (0.5) C: 12.7 yrs. (0.5) Mean years of education (SD): NA	(I) Ten schools *n* = NA (C) Eight schools *n* = NA Response rate: 84% Baseline: (I) *n* = 632 (C) *n* = 476 Lost to follow-up: 21% Randomisation	NA	8-month intervention (shorter than follow-up) Environmental: PA and diet (a) Curriculum: adaptation of the school curriculum to include 11 lessons in biology and PE promoting healthy lifestyle. School encouraged to include more PE classes (b) Canteen: school encouraged to make healthy changes	Mean BMI before-after Males Baseline to T1 (I) 18.2 (2.6) versus 18.6 (2.8) (C) 19.0 (2.9) versus 19.4 (2.9) (*p*=NS) Baseline to T2 (I) 18.2 (2.6) versus 19.1 (3.0) (C) 19.0 (2.9) versus 19.8 (3.0) (*p*=NS) Baseline to T3 (I) 18.2 (2.6) versus 19.4 (2.9) (C) 19.0 (2.9) versus 20.0 (2.7) (*p*=NS) Females Baseline to T1 (I) 19.0 (3.0) versus 19.5 (3.1) (C) 19.5 (3.4) versus 20.0 (3.5) (*p*=NS) Baseline to T2 (I) 19.0 (3.0) versus 19.9 (3.2) (C) 19.5 (3.4) versus 20.3 (3.4) (*p*=NS) Baseline to T3 (I) 19.0 (3.0) versus 20.2 (2.9) (C) 19.5 (3.4) versus 20.9 (3.6) (*p*=NS)
Naul et al. [48] (The Netherlands) “Healthy children in sound communities” (HCSC/gkgk)--a Dutch-German community-based network project.”	Quasiexperimental changes in BMI and fitness 1 yr. (cohort)	(I) Children (6–10 yrs.) from Dutch schools (C) Children (6–10 yrs.) from German schools located in the same Dutch-German border region	Gender: NA I: 6.96 yrs. (0.56) C: 7.24 yrs. (0.24) Mean years of education (SD): NA	(I) Thirteen schools *n* = NA (C) Six schools *n* = NA Response rate: NA Baseline: *n* = 744 Lost to follow-up: 25% No randomisation	Schools were selected from a sample of 39 primary schools that had implemented an intervention in their school	1st year (4-year intervention) Environmental: PA and diet (a) Curriculum: 3 hours/week of tailored PE, one hour of cross-curricular education per week with a focus on health, and nutrition, healthy-active school breaks (b) Teachers: training (health, PE, and nutrition) Toolbox (project homepage): lesson plans for PE (c) Parents: events for children and their parents e.g., cooking classes (d) School-wide: active commuting to school-walking school bus (e) Community: one-hour extra PA, facilitated by sport clubs, offered 2 afternoons/week. Training for coaches. (f) Supporting materials: project homepage	Mean BMI before-after (I) 16.3 (NA) versus 16.6 (NA) (*p*=0.001) (C)16.5 (NA) versus 16.7 (NA) (*p*=0.001) (*p* = significant for heavy overweight and obese children, NA)

SD: standard deviation; SES: socioeconomic status; PA: physical activity; BMI: body mass index; BP: blood pressure; yrs.: years; yr.: year; I: intervention; C: control; n: number; NA: not available; CVD: cardiovascular disease; NS: non-significant; NGO: non-governmental organizations; MONICA: multinational monitoring of trends and determinants in Cardiovascular disease; PE: physical education; II: intense direct intervention; CI: county-wide intervention; IDEFICS: the identification and prevention of dietary- and lifestyle-induced health Effects in children and infants approach. ^a^Additional references (e.g., design article) for further information on baseline data and design. ^b^Does not apply for mean age when all children are at the same grade (same age). ^c^Mean years of education or SES for schools where we refer to the years of education of the parents and not of the children or adolescents. ^d^High refers to high education as defined as university education [34, 42, 43, 65], or 13 years or more of education [56, 57].^e^Data presented only for BMI changes and not obesity prevalence changes. Data are presented by gender and intervention only if total data by intervention group are not available.

**Table 3:** Summary of key characteristics of setting-based interventions.

Setting	Study type	Total follow-up	Gender as percentage (%) of females	Outcome measures	Intervention components	Theory based	BMI change
Community-based, *n* = 7	Pre-post studies (no control), *n* = 2 Quasiexperimental, *n* = 5	3–8 years Cohort and cross-sectional samples, *n* = 4	46.0% to 57.2%. Female	CVD risk factors, *n* = 7 BMI change (Adults), *n* = 7	Multicomponent, *n* = 5 Individual component, *n* = 7 Diet, PA, and other risk factors, *n* = 6 PA only, *n* = 1 Worksite, *n* = 2 Schools and worksites, *n* = 1 Organised activities, *n* = 6 Awareness campaigns, *n* = 7 Food stores, *n* = 4 Infrastructure, *n* = 2 Policy (smoking) = 2 Capacity building = 1	Explicitly theory-based, *n* = 1 Mentioned theory only, *n* = 1	Positive, *n* = 2 Negative, *n* = 2 No effect, *n* = 3

Worksite-based, *n* = 3	Quasiexperimental, *n* = 3	1–2 years Cohorts *n* = 3	37.4% to 50.7% female (2/3) 100% male drivers, *n* = 1	CVD risk factors, *n* = 2 Body Mass composition, *n* = 1 BMI change (adults), *n* = 3	Multicomponent, *n* = 1 Single component, *n* = 1 Individual component, *n* = 2 Diet and PA, *n* = 3 Organised activities, *n* = 2 Awareness campaigns, *n* = 2 Canteens, *n* = 2	Theory-based and built upon a multilevel approach, *n* = 1	No effect, *n* = 3

School-based, *n* = 23	Cluster randomised, *n* = 9 Historical controls used, *n* = 1 Quasiexperimental, *n* = 13	4 months-8 years Cohort and cross-sectional samples, *n* = 1 Cross-sectional samples only, *n* = 1	39.7% to 60.1% female (22/23) 100% female, *n* = 1	Obesity, *n* = 12 Body composition, *n* = 6 CVD risk factors, *n* = 5 BMI change (children and adolescents), *n* = 23 Weight prevention was a secondary outcome, *n* = 5	Multicomponent, *n* = 14 Single component, *n* = 7 Diet, PA, and other risk factors, *n* = 5 Diet, PA, *n* = 9 PA only, *n* = 6 Diet only, *n* = 6 Components for the parents, *n* = 5 Components for the teachers, *n* = 2 Components for parents and teachers, *n* = 8 Curriculum changes, *n* = 19 Food provision, *n* = 9 School-wide, *n* = 5 Awareness campaigns, *n* = 4 Policy, *n* = 4 Community, *n* = 3 Capacity building, *n* = 2 School infrastructure, *n* = 2 Curriculum component only for increase in PA, *n* = 4 Actions or changes related to diet, *n* = 3	Explicitly theory-based using a multilevel approach; like intervention mapping and whole school participation, n = 10	Negative, *n* = 1 Positive, *n* = 4 No effect, *n* = 15 Positive for control group only, *n* = 1 No data available, *n* = 3

*n*: number; CVD: cardiovascular disease; BMI: body mass index; PA: physical activity; multicomponent studies: 3 or more components; positive: significantly lower increase in BMI; negative: significantly higher increase in BMI; no effect: no significant effect; positive for control group only: significant increase in BMI for control group only; no data available, *n* = 3.

**Table 4:** Quality assessment of setting-based interventions.

Study	Suitability of study design	Number of criteria met	Representativeness^b^	Randomisation	Comparability^c^	Credibility of data collection instruments	Attrition rate	Attributability to intervention^d^
*Community-based*								
Jenum et al. [39]	Category A	1	NO	NO	NO	YES	NO	NO
Jenum et al. [59]^a^								
Lupton et al. [45]	Category A	3	NA	NO	YES	YES	NO	YES
Kumpusalo et al. [42]	Category A	3	YES	NO	NO	YES	YES	NA
Kumpusalo et al. [65]^a^								
Isacsson et al. [37]	Category B	3	YES	^E^	—	YES	YES	—
Lingfors et al. [44]	Category B	2	NO	—	—	YES	YES	—
Weinehall et al. [56]	Category A/Category B	2	YES	NO	NA	YES	NO	NA
Brannstrom et al. [57]^a^								
Schuit et al. [52]	Category A	4	YES	NO	YES	YES	YES	NA
*Worksite-based*								
Engbers et al. [34]	Category A	4	NO	NO	YES	YES	YES	YES
Kwak et al. [43]	Category A	3	NO	NO	YES	YES	YES	NA
Hedberg et al. [36]	Category A	4	YES	NO	YES	YES	YES	YES
*School-based*								
Ask et al. [24]	Category A	3	NO (pilot study)	YES (out of two units)	YES	YES	NA	NA
Ask et al. [25]	Category A	4	NO	YES (out of three units)	YES	YES	YES	NA
Bere et al. [26]	Category A	3	YES	YES	NA	YES	NO	NA
Grydeland et al. [35]	Category A	4	NO	YES	YES	YES	YES	NA
Resaland et al. [51]	Category A	2	NO	NO	YES	YES	NO	NA
Bugge et al. [27]	Category A	2	NO	NO	YES	YES	NO	NA
Klakk et al. [40]	Category A	3	NO	NO	YES	YES	YES	NA
Puska et al. [50]	Category A	2	NO	NO	NA	YES	YES	NA
Magnusson et al. [46]	Category A	3	NO	YES	YES	YES	NO	NA
Elinder et al. [33]	Category A	2	NO	NO	NA	YES	YES	NA
Elinder et al. [66]^a^								
Marcus et al. [47]	Category A	2	NO	YES	NA	YES	NO	NA
Nyberg et al. [49]	Category A	4	NO	YES	YES	YES	YES	NA
De Henauw et al. [31]	Category A	2	NA	NO	NA	YES	YES	NA
Hense et al. [67]^a^								
Ahrens et al. [68]^a^								
Sollerhed and Ejlertsson [54]	Category A	3	NO	NO	YES	YES	YES	NA
Stenevi-Lundgren et al. [55]	Category A	3	NO	NO	YES	YES	YES	NA
Busch et al. [28]	Category A	2	NO	NO	YES	YES	NO	NA
Busch et al. [29]	Category C	2	NO (pilot study)	NO	NA	YES	NA	YES
de Greeff et al. [30]	Category A	3	NO	YES	YES	YES	NA	NA
Kocken et al. [41]	Category A	3	NO	YES	YES	YES	NO	NA
de Meij et al. [32]	Category A	3	NO	NO	YES	YES	YES	NO
Jansen et al. [38]	Category A	4	NO	YES	YES	YES	YES	NA
Singh et al. [53]	Category A	3	NO	YES	NO	YES	YES	NA
Naul et al. [48]	Category A	2	NO	NO	NO	YES	YES	NA

NA: not available. ^a^Additional references (e.g., design article) for further information on baseline data and design. ^b^For the studies on schools, representativeness referred to the schools as units and not to the children/students participating. ^c^Baseline characteristics description or matching, if baseline BMI was not mentioned we considered Na. ^d^Based on what is discussed or reported in the article, we did not consider NO in cases where I and C were in proximity, unless a possibility of contamination is discussed. ^e^Does not apply, no control group.
